# Pinocembrin suppresses oxidized low-density lipoprotein-triggered NLRP3 inflammasome/GSDMD-mediated endothelial cell pyroptosis through an Nrf2-dependent signaling pathway

**DOI:** 10.1038/s41598-022-18297-3

**Published:** 2022-08-16

**Authors:** Tong Wang, Hua Tian, Tianqi Pan, Shutong Yao, Huayun Yu, Yumei Wu, Shijun Wang

**Affiliations:** 1 College of Nursing, Shandong University of Traditional Chinese Medicine, Jinan, 250355 Shandong China; 2 College of Traditional Chinese Medicine, Shandong University of Traditional Chinese Medicine, No. 4655 Daxue Road, Changqing District, Jinan, 250355 Shandong China; 3 Key Laboratory of Atherosclerosis in Universities of Shandong, Institute of Atherosclerosis, Shandong Academy of Medical Sciences, Shandong First Medical University, Taian, 271000 Shandong China; 4 College of Basic Medical Sciences, Shandong Academy of Medical Sciences, Shandong First Medical University, Taian, 271000 Shandong China; 5 Pharmacy Department, Dongying District Hospital of Traditional Chinese Medicine, Dongying, 257000 Shandong China

**Keywords:** Cardiovascular diseases, Aortic diseases, Inflammation, Inflammasome

## Abstract

Pinocembrin (Pin) has been confirmed to exert anti-inflammatory and antiatherosclerotic effects. Here we have explored whether and how Pin would protect vascular endothelial cells against pyroptosis elicited by the exposure to oxidized low density lipoprotein (oxLDL). Our results showed that Pin preconditioning dose-dependently suppressed oxLDL-stimulated HUVEC injury and pyroptosis, which were manifested by improved cell viability, lower lactate dehydrogenase (LDH) levels and DNA damage as well as decreased expression of pyroptosis-related markers, such as NOD-like receptor pyrin domain-containing 3 (NLRP3), apoptosis-associated speck-like protein containing a caspase activation and recruitment domain (ASC), pro-Caspase-1, cleaved Caspase-1, N-terminus of Gasdermin D-N (GSDMD-N), pro-interleukins-1β (pro-IL-1β), IL-1β and inflammatory cytokines (IL-18 and IL-1β). All of the effects were similar to those of MCC950 (an NLRP3 inhibitor). As expected, Pin distinctly activated the Nuclear factor erythroid 2-related factor 2 (Nrf2) antioxidative signaling pathway assessed through increased expressions of Nrf2, heme oxygenase-1 (HO-1) and NAD(P)H quinone oxidoreductase 1 (NQO1). Furthermore, after transfection with small interfering RNA of Nrf2, the inhibitory effects of Pin on oxLDL-triggered NLRP3 inflammasome/GSDMD-mediated pyroptosis and oxidative stress in HUVECs were weakened. Additionally, Pin up-regulated Nrf2/HO-1 axis and down-regulated NLRP3 inflammasome/GSDMD-mediated pyroptosis signals in Apoe^−/−^ mice fed with high fat diet. These results contribute to the understanding of the anti-pyroptosis mechanisms of Pin and provide a reference for future research on the anti-atherosclerotic effect of Pin.

## Introduction

Atherosclerosis (AS) is a chronic inflammatory disease caused by the deposition of modified lipoproteins in the intima of arteries. Vascular endothelial cells (VECs) are the first defense component of blood vessel walls. VECs injury and dysfunction are the driving forces of AS development^1^. Oxidized low density lipoprotein (oxLDL)-induced VECs damage is a prerequisite for the cholesterol uptake by subintimal macrophages, inflammation, the migration and proliferation of vascular smooth muscle cells^2^.

Recently, several authors have observed that oxLDL can induce NOD-like receptor family pyrin domain-containing 3 (NLRP3) inflammasome-mediated pyroptosis in VECs^3,4^. Indeed, the NLRP3 inflammasome is highly expressed in the aorta of atherosclerotic patients and the level of expression is proportional to the severity of the disease^5,6^. After activation, NLRP3 recruits the adaptor apoptosis-associated speck-like protein containing a C-terminal caspase recruitment domain (ASC) and the effector Pro-Caspase-1 to form the NLRP3 inflammasome, which results in Caspase-1 maturation. Subsequently, Caspase-1 mediates the cleavage of gasdermin-D (GSDMD) and pro-interleukins (i.e. Pro-IL-18 and Pro-IL-1β) into GSDMD-N, IL-18 and IL-1β, respectively. GSDMD-N helps to generate the pores in membranes that allow IL-18 and IL-1β to be released into the cytoplasm, triggering an inflammatory response, a process known as NLRP3 inflammasome/GSDMD-mediated pyroptosis^7,8^, which is closely associated with endothelial injury and atherogenesis^9,10^. Consequently, inhibition of NLRP3 inflammasome/GSDMD-mediated pyroptosis may be an effective therapeutic strategy to protect VECs from injury in AS.

Several basic studies have revealed the protective effect of various flavonoids in AS by interfering with oxLDL-induced apoptosis of VECs^11–13^. However, there are few studies on their effects on oxLDL-challenged VEC pyroptosis during the development of AS. Pinocembrin (Pin), a natural flavonoid abundant in propolis and honey and other plants such as *Populus* and *Pinus heartwood*^14^, has multiple pharmacological effects, including anti-oxidative, anti-allergic, anti-apoptotic, anti-inflammatory, anti-microbial and anti-AS^15–17^. Notably, Pin inhibits NLRP3 inflammasome/GSDMD-mediated pyroptosis in lipopolysaccharide and bleomycin- induced acute lung injury, doxorubicin-induced cardiac dysfunction as well as hypoxia-induced neuroinflammation^18–20^. Qiang Su et al. verified that Pin protects endothelial cells of the human aorta from oxLDL-stimulated inflammation^21^. Therefore, it is worth investigating whether Pin also affects NLRP3 inflammasome/ GSDMD-mediated pyroptosis on oxLDL-challenged VECs and possible upstream molecular mechanisms.

Nuclear factor erythroid 2-related factor 2 (Nrf2), a cytoprotective and redox-sensitive transcription factor widely expressed in several cells and tissues, is referred to as the master regulator of antioxidative and cytoprotective genes^22^. Curiously, Nrf2 has been implicated in the pathogenesis of AS^23^. Some compounds, such as Zedoarondiol and euxanthone, prevent endothelial cells from oxLDL- triggered oxidative stress (OxS) by enhancing Nrf2 activation^24,25^. Therefore, Nrf2 is likely to be a promising target for protecting the endothelial cells in AS. Previous studies reported that Pin activates Nrf2 and subsequently promotes the transcription of endogenous antioxidants, including heme oxygenase-1 (HO-1) and NAD(P)H quinone oxidoreductase 1 (NQO1) in vivo and in vitro^26–28^. To our knowledge, the effect of Pin on this signaling pathway in oxLDL-stimulated endothelial cells has not been reported. Excitingly, some natural compounds, including flavonoids, can activate the Nrf2/HO-1 axis and thus block NLRP3 inflammasome/GSDMD-mediated pyroptosis in endothelial cell injury models induced by various stimuli, including oxLDL^3,29^. Hence, we have explored if Pin exerts protective effects on oxLDL-stimulated HUVECs and plaque endothelial cells in Apoe^−/−^ mice and assessed if NLRP3 inflammasome/GSDMD-mediated pyroptosis and Nrf2/HO-1 axis were implicated in the effects mediated by Pin.

## Results

### Cytotoxicity and pyroptosis in HUVECs stimulated by oxLDL

**Figure 1:**
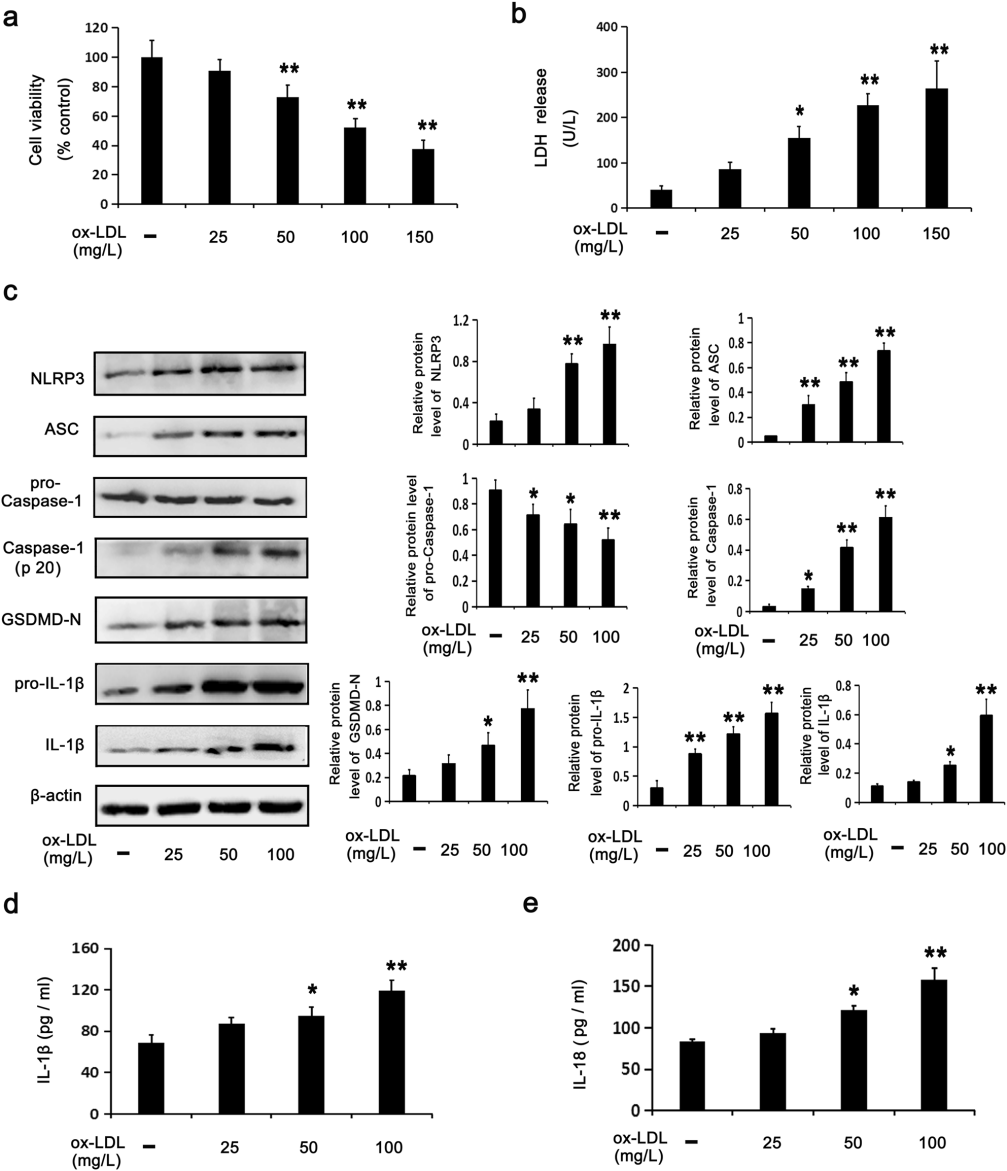
oxLDL stimulates cytotoxicity and pyroptosis among HUVECs. HUVECs were incubated with oxLDL (0–150 mg/L or 0–100 mg/L) for 24 h. CCK8 assay (**a**) and LDH assay (**b**) showing cell viability and LDH activity in media. (**c**) WB assays showing the protein levels of pyroptosis-related makers. ELISA assays for IL-1β (**d**) and IL-18 (**e**) in media. Data are described with mean ± SD of at least 3 different experiments. *P < 0.05; **P < 0.01 vs. cells in the control group.

CCK-8 and LDH assays were employed to detect cell viability and membrane disruption, respectively, according to our previously established oxLDL-stimulated HUVEC injury model.^30^ In this study we show that oxLDL stimulated cytotoxicity in HUVECs. Figure 1a,b show that, at increasing oxLDL concentrations (0–150 mg/L), cell viability was gradually reduced, while LDH release was increased. Besides, oxLDL dose-dependently induced pyroptosis in HUVECs. Figure 1c shows that oxLDL increased the levels of pyroptosis-related protein expression, namely NLRP3, ASC, Caspase-1 (P20), GSDMD-N, pro-IL-1β and IL-1β and decreased the protein level of pro-Caspase-1. Additionally, oxLDL increased IL-18 and IL-1β levels in medium (Fig. 1d,e). These results confirmed that oxLDL activated the NLRP3 inflammasome, GSDMD and pro-interleukins, resulting in the release of IL-18 and IL-1β. Considering that 100 mg/L oxLDL induced an approximate 50% reduction in cell viability compared to the control cells, this concentration was adopted in the following experiments. Based on the above results, it was reinforced that the oxLDL-stimulated cytotoxicity and pyroptosis of HUVECs is a stable injury model.

### Pin alleviates oxLDL-stimulated injury among HUVECs

**Figure 2:**
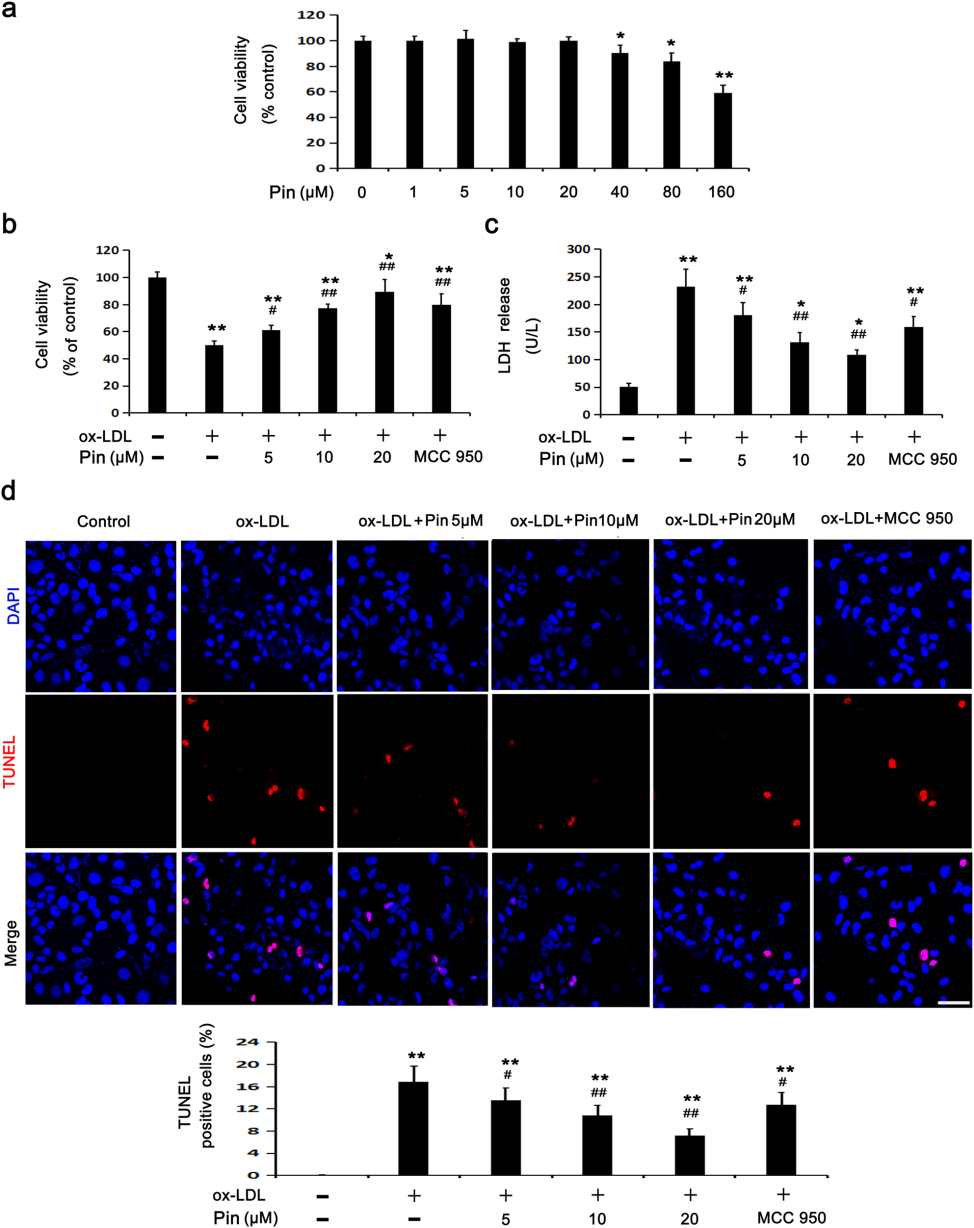
Role of Pin in oxLDL-challenged HUVECs. HUVECs were incubated with Pin (0–160 μM) for 24 h. (**a**) Cell viability was detected through the CCK8 assay. Cells were pretreated with Pin (5, 10, 20 μM) or MCC950 (1 μM) for 2 h before incubated with oxLDL (100 mg/L) for 24 h. CCK8 assay (**b**) and LDH assay (**c**) were adopted to assess cell viability and LDH level in media, respectively. (**d**) TUNEL assay was used to observe cell DNA damage. Red: DNA damaged cells; Blue: DAPI. Quantitative determination of TUNEL-positive cells is shown in the graphs. Scale bar: 50 µm; Data are described with mean ± SD for at least 3 different experiments. *P < 0.05; **P < 0.01 vs. control cells; ^#^*P* < 0.05; ^##^*P* < 0.01 vs. oxLDL-challenged cells.

To identify the ideal concentration of Pin in the following experiments, HUVECs were incubated with varying concentrations of Pin from 0 to 160 µM (Fig. 2a). Cell viability did not change significantly until to 20 µM Pin, but decreased gradually at 40–160 µM Pin, when compared to the control cells (0 µM Pin). Therefore, 5, 10 and 20 µM Pin were used in the following experiments.

oxLDL treatment reduced cell viability and enhanced LDH leakage (Fig. 1a,b). However, Pin (5, 10 and 20 μM) pretreatment showed a dose-dependent inhibitory effect on the above phenomena. MCC950, a widely used NLRP3 inhibitor, also abolished oxLDL-stimulated injury on HUVECs (Fig. 2b,c). To study the protective role of Pin in oxLDL-stimulated HUVECs, a TUNEL assay was performed to assess DNA damage. After 24 h of oxLDL treatment, the DNA damage in HUVECs was markedly enhanced compared to the control cells, whereas preincubation with Pin dose-dependently reduced the cell DNA damage. In parallel, MCC950 also inhibited the DNA damage in HUVECs exposed to oxLDL (Fig. 2d). We found that oxLDL resulted in HUVECs damage by activating NLRP3 inflammasome and the downstream pyroptosis-related signaling molecules, whereas Pin inhibited the damage in a manner similar to MCC950, suggesting that Pin may reduce HUVECs damage through suppressing NLRP3 inflammasome-mediated pyroptosis.

### Pin restricts NLRP3 inflammasome/GSDMD-mediated pyroptosis in HUVECs challenged by oxLDL

**Figure 3:**
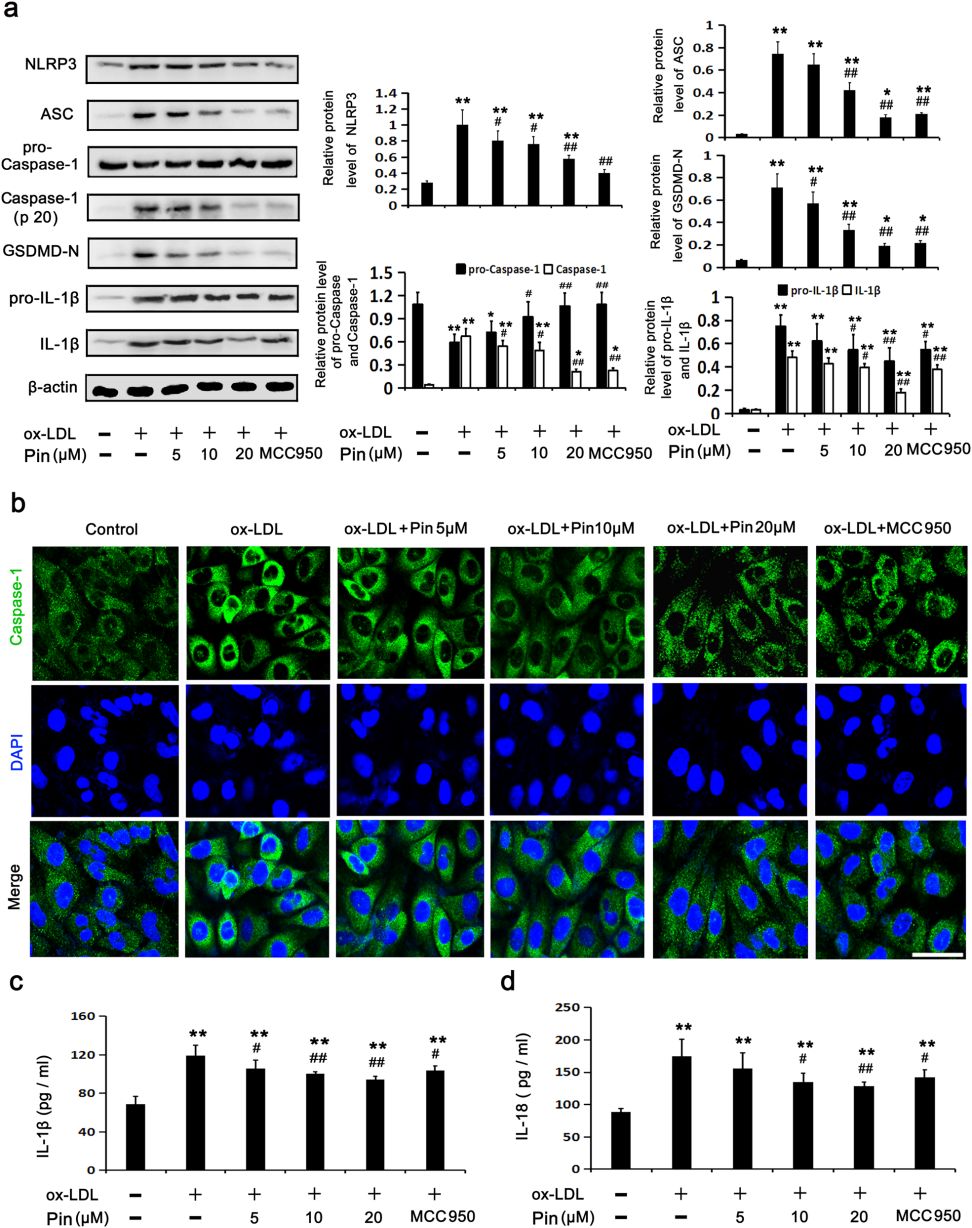
Role of Pin in oxLDL-triggered pyroptosis in HUVECs. HUVECs were pretreated with Pin (5, 10, 20 μM) or MCC950 (1 μM) for 2 h and incubated with oxLDL (100 mg/L) for 24 h. (**a**) WB showing the protein levels of pyroptosis-related makers. (**b**) Typical immunofluorescence images showing Caspase-1 expression in the cytoplasmic region. Green: Caspase-1; Blue: DAPI. Scale bar = 50 µm. (**c,d**) Showing IL-1β and IL-18 levels in the media, assayed through ELISA. Data are described with mean ± SD for at least 3 different experiments. *P < 0.05; **P < 0.01 vs. control cells; ^#^P < 0.05; ^##^P < 0.01 vs. oxLDL-challenged cells.

Consistent with the effects of MCC950, Pin reduced the injury of HUVECs challenged by oxLDL (Fig. 2). Thus, we hypothesized that suppression of the NLRP3 inflammasome/GSDMD-mediated pyroptosis pathway may be a potential mechanism through which Pin could reverse the oxLDL-induced injury. We found that the NLRP3 inflammation/GSDMD-mediated pyroptosis pathway was dramatically activated by oxLDL, which was evidenced by increased protein expression levels of NLRP3, ASC, Caspase-1 (P20), GSDMD-N, pro-IL-1β and IL-1β and decreased protein expression levels of pro-Caspase-1 among HUVECs (Fig. 1). However, similar to MCC950, Pin pretreatment significantly abolished the up-regulation of signaling molecules of NLRP3 inflammasome/GSDMD-mediated pyroptosis pathway triggered by oxLDL. This was especially evident at 20 μM Pin (Fig. 3a). Fluorescence staining of Caspase-1 was performed to further estimate Caspase-1-dependent pyroptosis triggered by oxLDL. Results showed that, like MCC950, Pin pretreatment alleviated the accumulation of Caspase-1 fluorescence in oxLDL-treated HUVECs, this effect was more evident at 20 μM Pin (Fig. 3b). Figure 3c,d show that pretreatment with various concentrations of Pin, particularly 20 μM, suppressed oxLDL-stimulated increases in extracellular IL-18 and IL-1β. These results depict that Pin could restrict NLRP3 inflammasome activation, the cleavage of GSDMD and subsequent release of IL-18 and IL-1β into the medium, similar to the effects observed using MCC950.

### Pin promotes the activation of the Nrf2/HO-1 axis in HUVECs challenged by oxLDL

**Figure 4:**
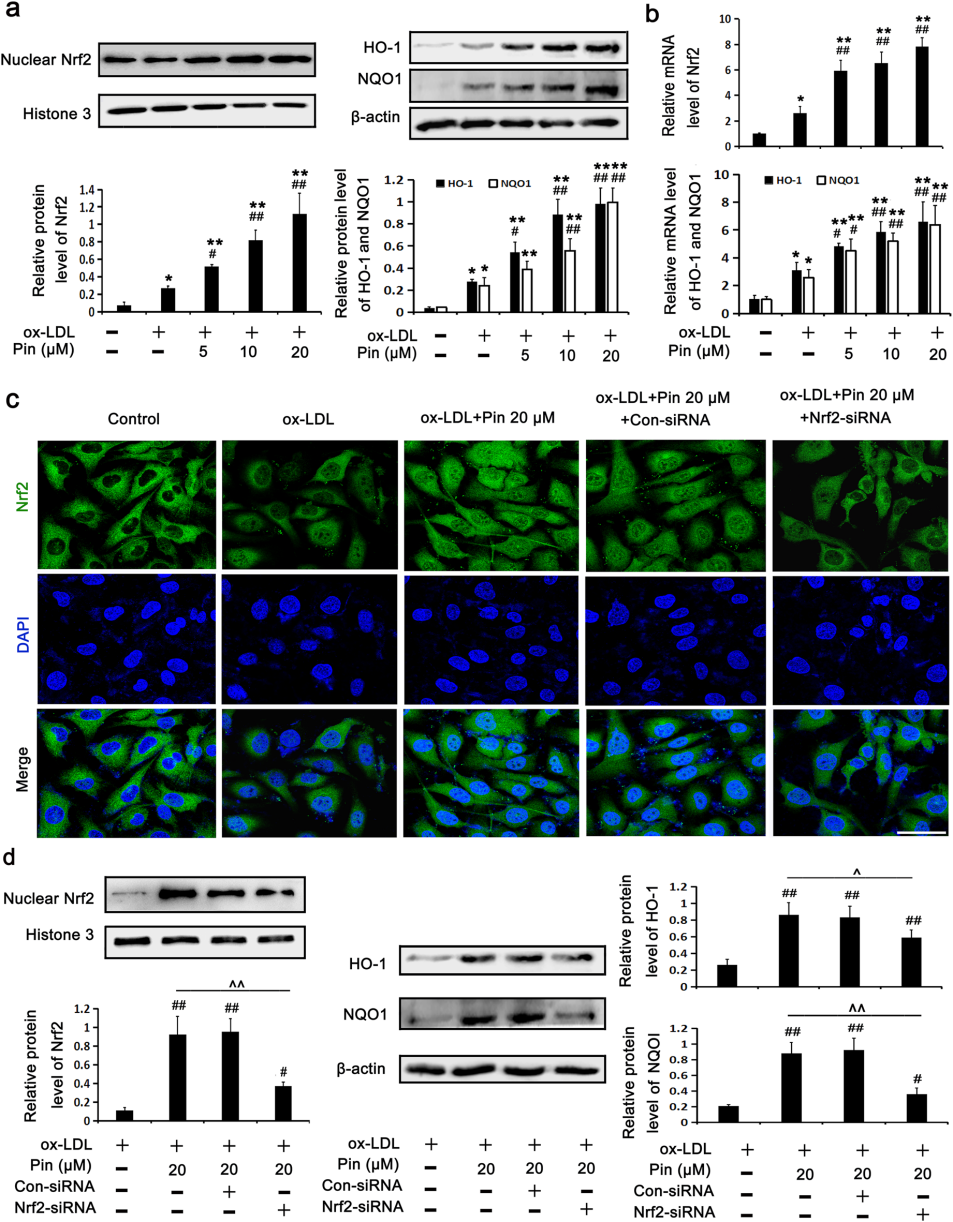
Role of Pin in the Nrf2/HO-1 axis in oxLDL-challenged HUVECs. HUVECs were preincubated with Pin (5, 10, 20 μM) for 2 h, followed by the incubation with 100 mg/L oxLDL. (**a**) WB assays showing protein levels in the Nrf2/HO-1 axis. (**b**) RT-PCR assays showing mRNA levels in the Nrf2/HO-1 axis. Cells were transfected with Con-siRNA or Nrf2-siRNA, and then incubated with oxLDL (100 mg/L) for an additional 24 h with or without 20 µM Pin pretreatment. (**c**) Typical immunofluorescence images showing Nrf2 nuclear translocation. Green: Nrf2; Blue: DAPI. Scale bar = 50 µm. (**d**) WB assays showing protein levels of the Nrf2 signaling pathway. Data are described with mean ± SD of at least 3 different experiments. *P < 0.05; **P < 0.01 vs control cells; ^#^*P* < 0.05; ^##^*P* < 0.01 vs. oxLDL-challenged cells. ^^^*P* < 0.05, ^^^^*P* < 0.01, compared between the marked groups.

Given that Pin was shown to activate the Nrf2/HO-1 axis in previous studies^26–28^, we speculated that this might be a feasible mechanism through which Pin protects HUVECs from pyroptosis induced by oxLDL. To confirm this hypothesis, we analyzed protein expression levels of proteins within the Nrf2/HO-1 axis. As described in Fig. 4a,b, oxLDL slightly increased Nrf2 nuclear protein expression, NQO1 and HO-1 protein expression and also increased their mRNA levels compared with the control cells. Excitingly, incubation with Pin before exposure with oxLDL caused a further elevation of Nrf2 nuclear protein expression and HO-1 and NQO1 protein expressions in a dose dependent maner when compared to results found in the oxLDL group. These results corroborate that the mild stimulation by oxLDL could not sufficiently activate the Nrf2/HO-1 axis so as to counteract oxLDL-triggered pyroptosis in HUVECs. So we expected that Pin could facilitate the activation of the Nrf2/HO-1 axis to block oxLDL-triggered HUVEC pyroptosis.

To further investigate the role of Pin in the Nrf2/HO-1 axis among endothelial cells exposed to oxLDL, HUVECs were first transfected with Nrf2 siRNA, followed by oxLDL exposure with or without Pin. As shown in Fig. 4c, immunofluorescence staining showed that oxLDL stimulated a mild Nrf2 nuclear translocation compared with the control cells. Additionally, Pin pretreatment promoted a clear nuclear translocation of Nrf2 compared with oxLDL exposure alone, which was blocked by Nrf2 siRNA. Indeed, Nrf2 siRNA abrogated the up-regulation of Nrf2, HO-1 and NQO1 protein levels promoted by Pin pretreatment in oxLDL-exposed HUVECs (Fig. 4d).

### Nrf2 siRNA blocks Pin-inhibited OxS in HUVECs challenged by oxLDL

**Figure 5:**
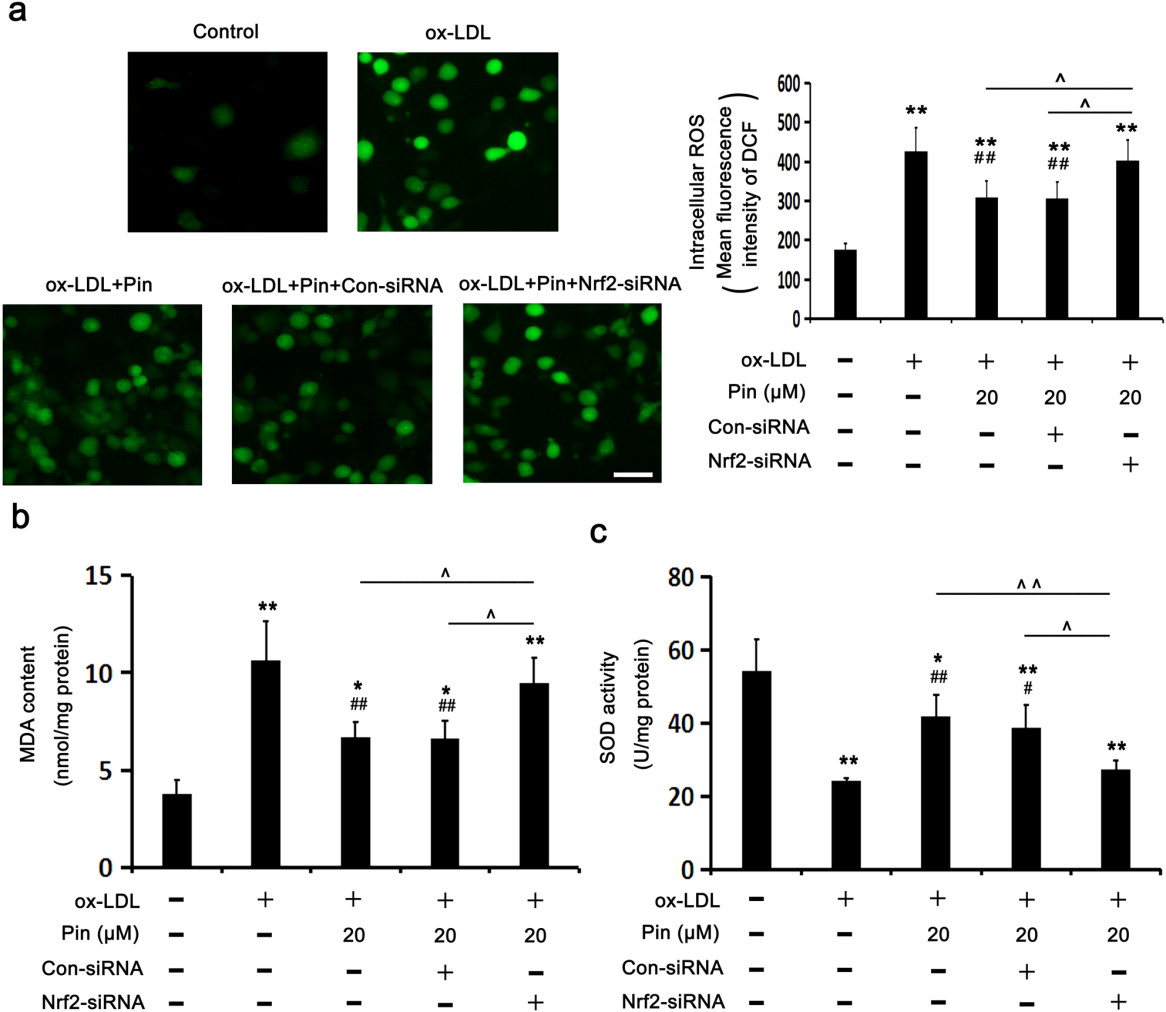
Role of Pin in OxS depends on Nrf2 in oxLDL-challenged HUVECs. HUVECs were transfected with con-siRNA or Nrf2-siRNA, pretreated with or without 20 µM Pin for 2 h and then incubated with 100 mg/L oxLDL for an additional 24 h. (**a**) DCF fluorescence was quantified using a flow cytometer to measure the levels of intracellular ROS and the representative fluorescence images depicting ROS staining. Scale bar = 50 µm. (**b,c**) The production of MDA and SOD activity in HUVECs, respectively. Data are described with mean ± SD of at least 3 different experiments. *P < 0.05; **P < 0.01 vs. control cells; ^#^*P* < 0.05; ^##^*P* < 0.01 vs. cells challenged by oxLDL. ^^^*P* < 0.05, ^^^^*P* < 0.01, compared between the marked groups.

Pin was shown to ameliorate OxS through the stimulation of Nrf2/HO-1 axis in cellular and animal models^26–28^. Therefore, we have investigated if the inhibitory role of Pin in oxLDL-triggered HUVEC pyroptosis could be attributed to OxS reduction. Intracellular ROS were determined using DCHF (a fluorescent probe), while the activity of SOD (an antioxidant enzyme) and the production of MDA (a lipid peroxidation marker). Figure 5 showed that HUVECs challenged by oxLDL displayed significantly enhanced intracellular ROS and MDA levels, meanwhile, decreased the activity of SOD compared to the control cells. As expected, pretreatment with Pin prominently decreased the oxLDL-generated ROS and MDA levels and recovered the SOD activity. Nevertheless, the inhibition of oxLDL-induced OxS by Pin was dependent on Nrf2, as evidenced by the increase of ROS and MDA levels as well as the reduction of SOD activity after Nrf2-siRNA transfection. Based on the above results, it was evidenced that Nrf2 mediates the antioxidant role of Pin.

### Nrf2 siRNA abrogates the inhibitory role of Pin in NLRP3 inflammasome/ GSDMD-mediated pyroptosis in HUVECs challenged by oxLDL

**Figure 6:**
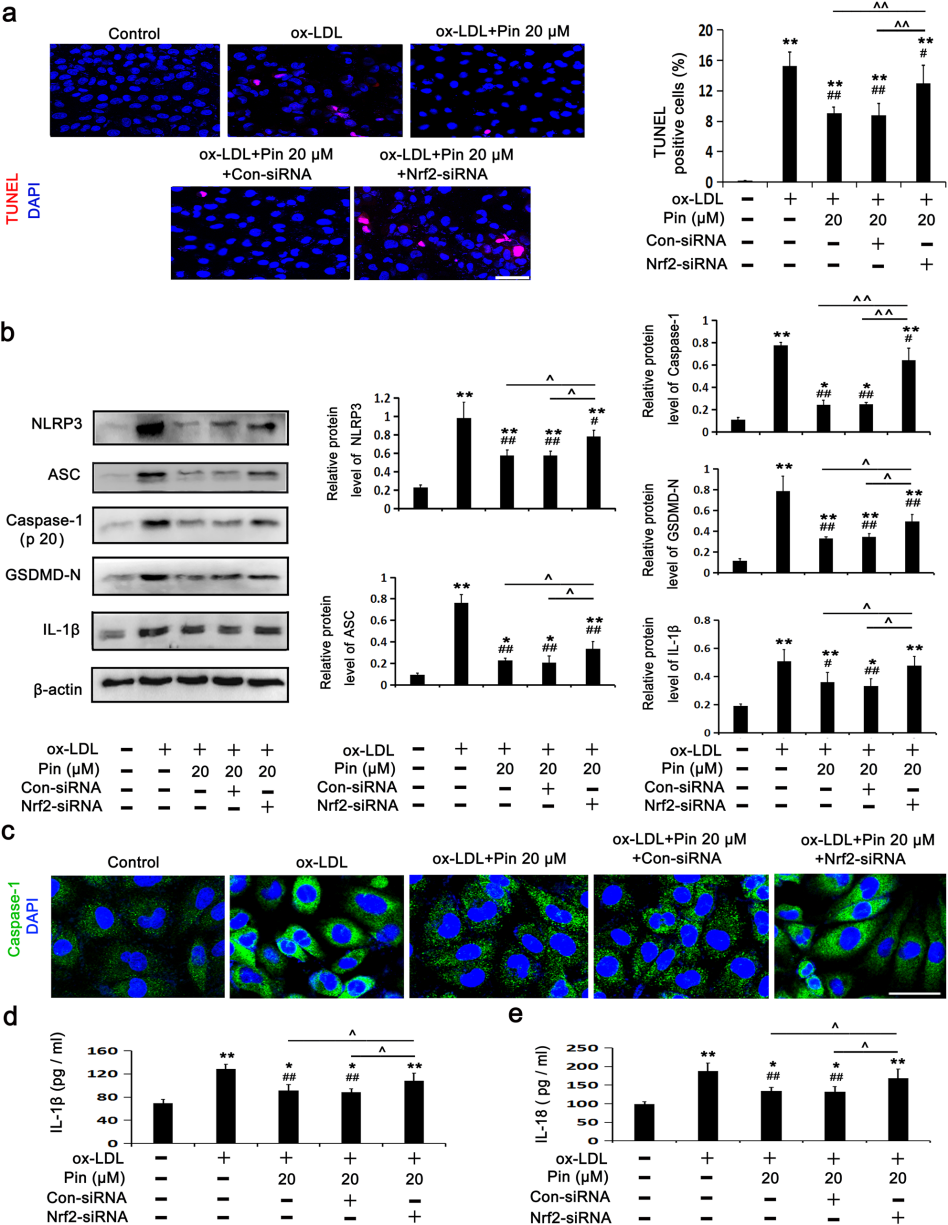
Role of Pin in pyroptosis depends on Nrf2 in oxLDL-challenged HUVECs. After transfection with con-siRNA or Nrf2-siRNA, HUVECs were pretreated with or without 20 µM Pin for 2 h, and then incubated with 100 mg/L oxLDL for an additional 24 h. (**a**) TUNEL assay was used to observe cell DNA damage. Red: DNA damaged cells; Blue: nuclei. Quantitative determination of TUNEL-positive cells is shown in the graphs. Scale bar: 50 µm; (**b**) WB assays showing protein levels of pyroptosis-related markers. (**c**) Typical immunofluorescence images showing Caspase-1 expression in the cytoplasmic region. Green: Caspase-1; Blue: DAPI. Scale bar = 50 µm. (**d,e**) Showing IL-1β and IL-18 levels in the media measured by ELISA. Data are described with mean ± SD of at least 3 different experiments. *P < 0.05; **P < 0.01 vs. control cells; ^#^*P* < 0.05; ^##^*P* < 0.01 vs. cells challenged by oxLDL. ^^^*P* < 0.05, ^^^^*P* < 0.01 compared between the marked groups.

To further illuminate the relationship between the Nrf2/HO-1 axis and NLRP3 inflammasome/GSDMD-mediated pyroptosis in the process of cell protection by Pin, we investigated the possibility of Pin in alleviating NLRP3 inflammasome-mediated pyroptosis of cells challenged by oxLDL through the Nrf2/HO-1 pathway. The results revealed that transfection with Nrf2-siRNA, but not Con-siRNA, prevented the Pin-exerted protection against cell DNA damage (Fig. 6a), as evidenced by the increase in protein expression levels of NLRP3, Caspase-1 (P20), ASC, GSDMD-N and IL-1β (Fig. 6b), the accumulation of Caspase-1 fluorescence (Fig. 6c) and the release of IL-18 (Fig. 6d) and IL-1β (Fig. 6e) among oxLDL-challenged HUVECs. These results suggest that the inhibitory effect of Pin on pyroptosis was mediated by the Nrf2/HO-1 axis. Moreover, these findings revealed that the Nrf2/HO-1 axis, activated by Pin, acted as an upstream signaling pathway to inhibit downstream NLRP3-mediated pyroptosis in HUVECs exposed to oxLDL.

### Pin promotes Nrf2/HO-1 axis and inhibits NLRP3 inflammasome/GSDMD-mediated pyroptosis in Apoe^−/−^ mice

**Figure 7:**
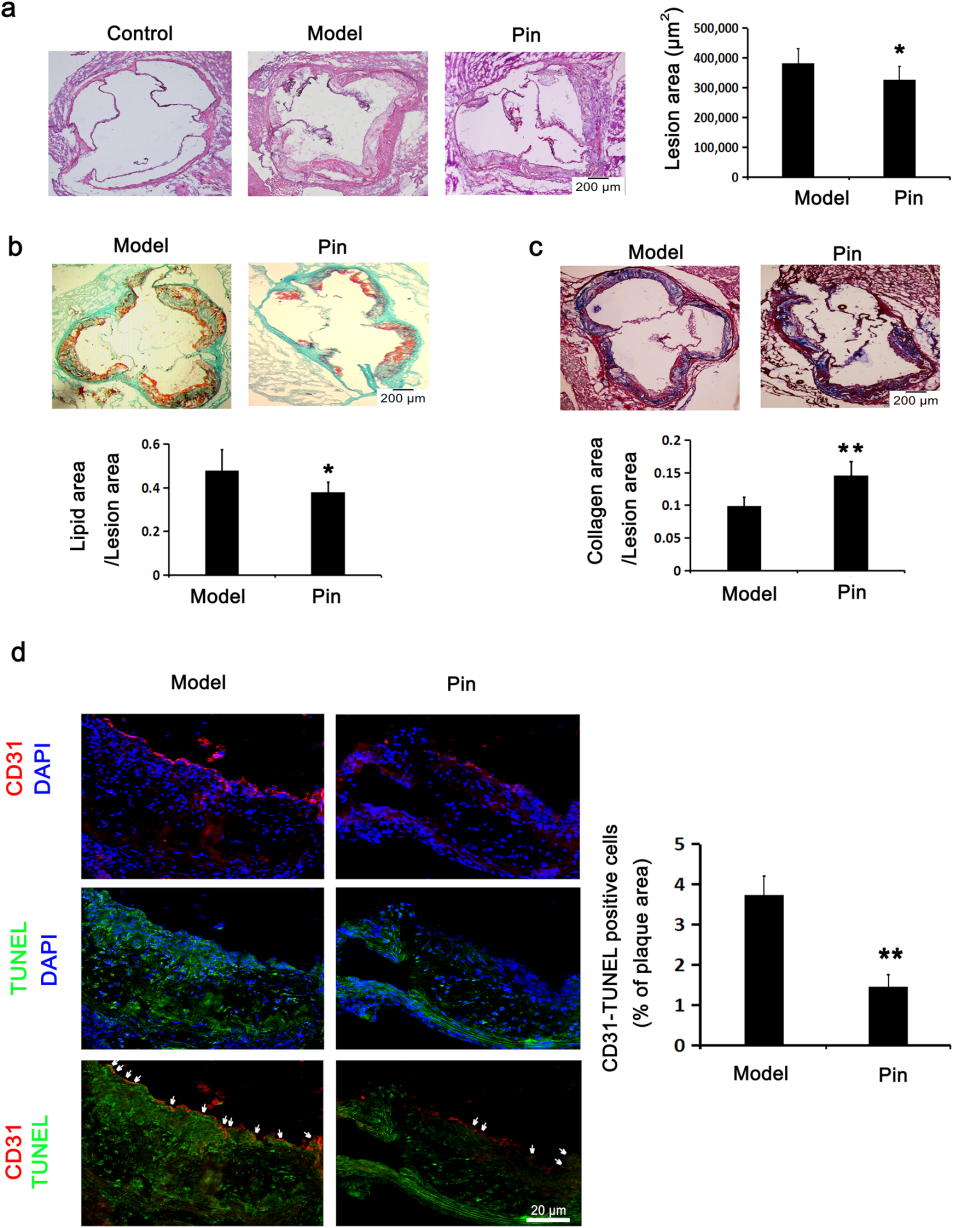
Role of Pin in atherosclerotic lesions and CD31-TUNEL-positive cells in Apoe^–/–^ mice. The atherosclerotic lesion model of Apoe^−/−^ mice fed a high-fat-diet was established and then intragastrically administered with the vehicle (model group) and pinocembrin (Pin group, 20 mg/kg), once daily for continuously 14 weeks. In addition, a normal chow diet was fed to male C57BL/6 mice as the background control of Apoe^−/−^ mice. (**a**) HE-stained aortic root cross-sections to examine the atherosclerotic lesion formation. Scale bar = 200 µm. (**b**) Lipid content stained by oil O red and bright green. Scale bar = 200 µm. (**c**) Collagen content stained by Masson. Scale bar = 200 µm. (**d**) Representative fluorescence micrographs depicting the CD31 and TUNEL staining images for endothelial cell with DNA damage in the aortic roots. Red: CD31; Green: TUNEL; Blue: nuclei. Scale bar = 20 µm. Data are described with mean ± SD of at least 3 different experiments. *P < 0.05; **P < 0.01 vs. model group.

**Figure 8:**
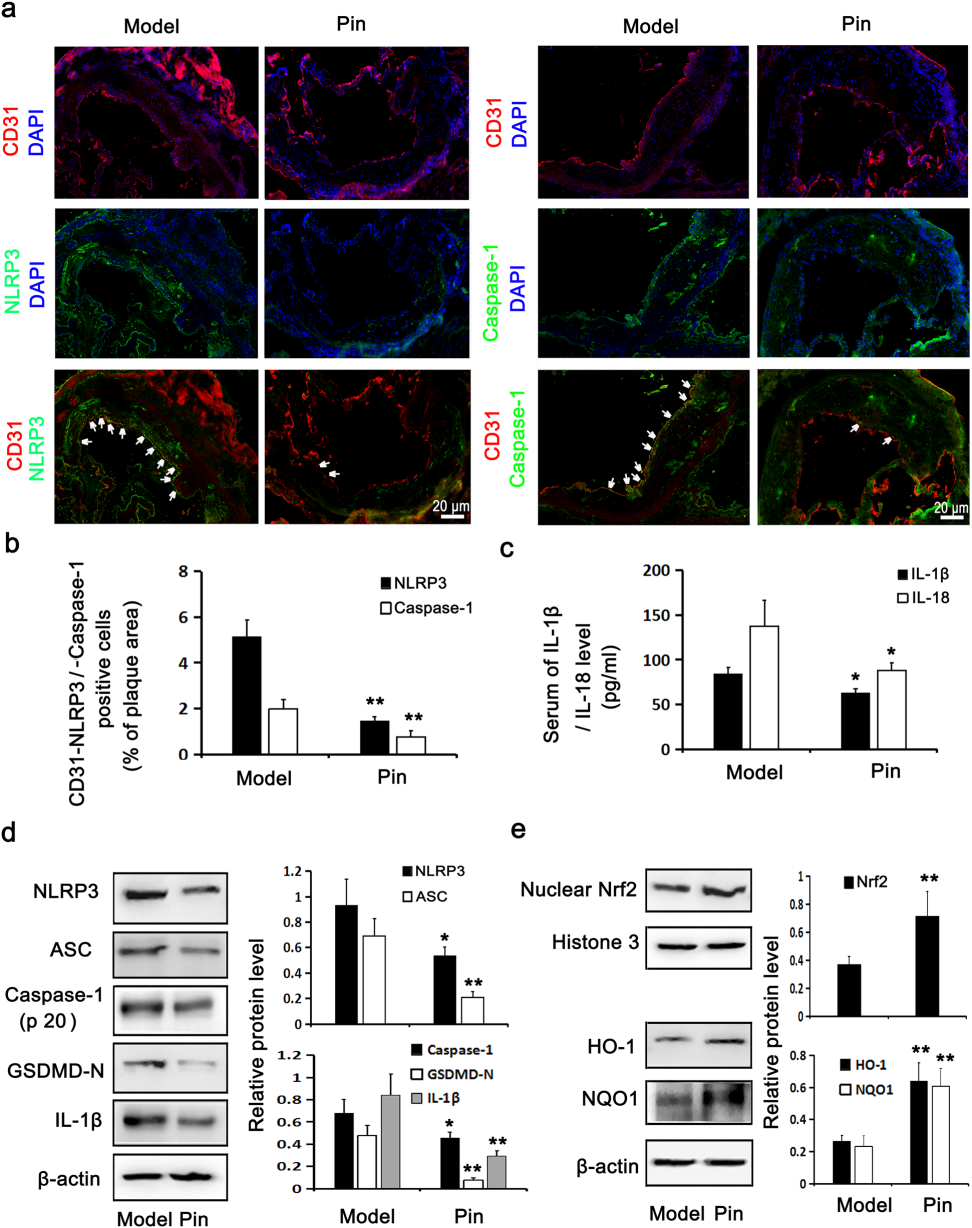
Role of Pin in the expressions of pyroptosis-related molecules and Nrf2/HO-1 axis in Apoe^–/–^ mice. The immunofluorescence stained images and quantitative analysis of CD31-NLRP3 and CD31-Caspase-1 positive cells (white arrow) in the aortic roots were displayed on (**a,b**). Red: CD31; Green: NLRP3 and Caspase-1; Blue: nuclei. Scale bar = 20 µm. (**c**) Showing IL-1β and IL-18 levels in the serum, assayed through ELISA. WB assay showing the protein expression levels of pyroptosis-related (**d**) and Nrf2/HO-1 axis-related molecules (**e**) in the aortic arches. The data are described as the mean ± SD of at least 3 different experiments. *P < 0.05; **P < 0.01 vs. model group.

To evaluate the antiatherosclerotic function of Pin in vivo, an experimental atherosclerotic mice model was established using Apoe^−/−^ mice. As described in Fig. 7, compared with the vehicle-treated model group, Pin administration for 12 weeks obviously decreased the atherosclerotic lesion areas, lipid content and the DNA damage in endothelial cells as well as increased the collagen content in aortic root plaques from Apoe^−/−^ mice. As shown in Fig. 8, Pin treatment significantly attenuated the NLRP3 inflammasome/GSDMD-mediated pyroptosis as assessed by the reduced NLRP3 and Caspase-1 (P20) expression in the endothelial cells of aortic root plaques, the decreased protein expression of NLRP3, ASC, Caspase-1(P20), GSDMD-N and IL-1β in aortic arches and the lowered level of IL-18 and IL-1β in serum compared with the model group. Moreover, Pin remarkably up-regulated Nrf2, HO-1 and NQO1 protein expression in aortic arches. These data suggest that Pin may also suppress the NLRP3 inflammasome/GSDMD-mediated pyroptosis and the DNA damage in endothelial cells within atherosclerotic lesions in Apoe^−/−^ mice by facilitating Nrf2/HO-1 axis.

## Discussion

Numerous clinical and experimental data confirm that endothelial inflammation is key to the development of AS^31^. Multiple factors such as excessive OxS, modification of lipoproteins, immune response, and inflammation can contribute to VEC dysfunction, which is an early event of AS^32^. Endothelial dysfunction caused by oxLDL is mainly due to its ability to reduce endothelial nitric oxide synthesis, increase levels of adhesion molecules and secretion of inflammatory cytokines as well as increase cell permeability, all of which lead to AS formation and subsequent clinical complications^33^.

The flavonoid Pin has been reported to suppress the expression levels of vascular adhesion molecules (E-selectin, vascular cell adhesion molecule 1 and intercellular cell adhesion molecule-1) and cytokines (tumor necrosis factor-α, IL-8, and IL-1β)^21^. PIN also antagonizes oxLDL-induced ROS generation by inhibiting mitogen- activated protein kinase (MAPK) and nuclear factor NF-kappaB (NF-κB) pathways^21^. Additionally, Pin is protective against fibrillar Aβ_1−40_-induced human brain microvascular endothelial cells injury via blocking MAPK and NF-κB pathways^34^. It has been observed that the NLRP3 inflammasome is critical in the pathological progression of various cardiovascular diseases including AS^35^. Furthermore, it has been proven that activation of the NLRP3 inflammasome and subsequent endothelial cell pyroptosis often lead to inflammation, which may be relevant for AS^36^. Extensive reports have confirmed that oxLDL triggers pyroptosis in HUVECs^3,4,37^. Consistently, we observed that oxLDL exposure decreased the viability of HUVECs, increased the expression of proteins related to NLRP3-inflammasome/GSDMD-mediated pytoptosis, including NLRP3, ASC, Caspase-1 (P20), GSDMD-N, pro-IL-1β, and IL-1β and decreased pro-Casepase-1, meanwhile, promoted the release of LDH, IL-18 and IL-1β. All these phenomena have been identified as the common features of pyroptosis. These results suggest that oxLDL-triggered pyroptosis relies on the activation of the NLRP3 inflammasome complex as well as GSDMD-N. Intriguingly, Pin was shown to inhibit the TLR4-NF-κB-NLRP3 inflammasome axis in RAW264.7 cells, J774A.1 cells and in mouse lung treated with lipopolysaccharide and bleomycin^18^. Furthermore, Pin alleviates myocardial cell pyroptosis by the activation of the Nrf2/Sirt3 axis, thereby protecting the heart against doxorubicin-induced cardiotoxicity^19^. Apart from that, Pin was described to suppress the formation of the NLRP3 inflammasome and enhance BCL2-interacting protein 3-mediated mitophagy in the hippocampus of mice subjected to intermittent hypoxia^20^. However, to date, few studies have investigated whether Pin can inhibit oxLDL-induced endothelial pyroptosis. Our study showed that Pin pretreatment significantly reduced oxLDL-stimulated injury, as evidenced by improved cell viability and reduced LDH leakage and DNA damage, similar to the results obtained using MCC950 (an antagonist of NLRP3). Additionally, Pin remarkably inhibited oxLDL-induced pyroptosis, since cells pretreated with this flavonoid presented reduced the protein levels of NLRP3 inflammasome/GSDMD-mediated pyroptosis pathway-related molecules and decreased Caspase-1 fluorescence accumulation as well as the release of inflammation mediators, similar to the anti-pyroptosis effect of MCC950. Altogether, the inhibitory effect of Pin on oxLDL-triggered HUVEC pyroptosis was correlated to the suppression of NLRP3-inflammasome and GSDMD pathways.

Kelch-like ECH-associated protein 1 (Keap1) is the endogenous inhibitor of Nrf2. At basal conditions, Nrf2 associates with Keap1 in the cytoplasm. OxS is implicated in the pathogenesis of various diseases including AS, which lead to the dissociation of Nrf2 from Keap1 and the translocation into the nuclei. Nrf2 then binds to antioxidant response elements and induces the transcription of antioxidants, of which HO-1 and NQO1 play very important antioxidant and anti-inflammatory roles in AS^38,39^. Studies using flavonoid compounds such as dihydromyricetin and genistein have demonstrated their ability to abrogate the damage to oxLDL-stimulated endothelial cells through activating the Nrf2 signaling pathway^40,41^. In this regard, Pin was shown to improve cardiac function and remodeling in rats with post-infarct heart failure. Pin was also shown to inhibit liver OxS and inflammation induced by carbon tetrachloride in vivo and protect SH-SY5Y cells from H_2_O_2_ damage through ROS scavenging and activation of the Nrf2/HO-1 axis^26–28^. Nevertheless, few studies have assessed if Pin upregulates the Nrf2/HO-1 axis to attenuate oxLDL-stimulated endothelial cell damage and pyroptosis. We have herein revealed that Pin further activated the Nrf2/HO-1 axis, as evidenced by increased protein and mRNA levels of Nrf2, HO-1 and NQO1 and promoted nuclear translocation of Nrf2 in oxLDL-exposed HUVECs. However, Pin-activated Nrf2/HO-1 axis was abolished through the transfection with Nrf2-siRNA, as evidenced by the decreased Nrf2 nuclear translocation and protein expresssion levels of the Nrf2/HO-1 axis-related molecules. Interestingly, the anti-pyroptotic effects of Pin in oxLDL-exposed HUVECs were weakened by Nrf2-siRNA transfection as determined by the increased cell DNA damage, upregulated expression of NLRP3 inflammasome/GSDMD-mediated pathway-related proteins and fluorescence accumulation of Caspase-1 as well as the release of interleukins. To sum up, our findings indicated that Pin could attenuate NLRP3 inflammasome/GSDMD-mediated HUVEC pyroptosis triggered by oxLDL through the upregulation of Nrf2/HO-1 axis.

Activation of the NLRP3 inflammasome requires two types of signals: a priming signal that upregulates NLRP3, Pro-IL-18 and Pro-IL-1 β expression, and another signal that assembles and activates the NLRP3 inflammasome. The priming signal is required for the NLRP3 inflammasome activation^42^. NF-κB activation is pivotal to upregulate NLRP3, Pro-IL-18 and Pro-IL-1 β during NLRP3 inflammasome priming. Several stimuli, including ROS inducers, TLR ligands, and inflammatory mediators have been shown to activate NF-κB^43^. Numerous reports have corroborated that oxLDL-induced OxS is mainly caused by excessive ROS generation and reduced antioxidant enzyme activity in HUVECs^44^. ROS acts primarily as a priming signal that upregulates NLRP3, Pro-IL-18 and Pro-IL-1 β expression by activating NF-κB^45^. ROS also acts as an activation signal to trigger NLRP3 inflammasome assembly and activation^46^. However, it remains controversial if ROS provides only priming or activatory signals in the NLRP3 inflammasome^47,48^. Taken together, there is growing evidence that ROS is a positive regulator of the NLRP3 inflammasome. Cumulative evidence has indicated that ROS and NF-κB are involved in NLRP3 inflammasome/GSDMD-mediated pyroptosis in endothelial cells, macrophages, human peripheral blood mononuclear cells and pigmented epithelial cells exposed to oxLDL^3,49–51^. Intriguingly, Nrf2 regulates antioxidant genes to ensure cell survival against OxS. In parallel, Nrf2 also inhibits NLRP3 activation by restricting ROS production^52^. A large number of reports, including ours, have proved that OxS caused by oxLDL is mainly due to (1) the overproduction of ROS derived from nicotinamide adenine dinucleotide phosphate oxidase and (2) the decrease of antioxidant enzyme activity^30^. We reported here that Pin pretreatment was protective against oxLDL-induced OxS in HUVECs in a mechanism that involved the inhibition of ROS generation, reduced SOD activity and increased MDA content. After transfection with siRNA for Nrf2-1, the anti-OxS functions exerted by Pin were greatly inhibited. Our data suggested that Pin may block OxS in oxLDL-stimulated endothelial cells by upregulating Nrf2/HO-1 axis, thereby inhibiting the priming and activation of NLRP3 inflammasome/GSDMD- mediated pyroptosis and subsequent cell damage. Furthermore, the results of animal experiments revealed that treating Apoe^−/−^ mice with Pin suppressed atherosclerotic palque formation and endothelial DNA damage. Moreover, Pin inhibited NLRP3 inflammasome/GSDMD-mediated pyroptosis signals and up-regulated Nrf2/HO-1 axis.

In conclusion, this study demonstrated that Pin was protective against NLRP3 inflammasome/GSDMD-mediated pyroptosis of oxLDL-stimulated HUVECs by upregulating the Nrf2/HO-1 axis. Further experiments revealed that Pin also suppressed ROS generation by promoting the Nrf2/HO-1 axis, which may contribute to inhibit the priming, assembly, and activation of NLRP3 inflammasome, and thereby alleviating oxLDL-triggered pyrophosis. Moreover, we also observed that Pin inhibited atherosclerotic palque formation and endothelial cell damage which may caused by NLRP3 inflammasome/GSDMD-mediated pyroptosis while activating Nrf2/HO-1 axis in atherosclerotic model mice. AS is known to be an inflammatory disease involving endothelial dysfunction, therefore, our study provides novel insights into the development of Pin as a therapeutic strategy for AS.

## Materials and methods

### Reagents

Pin and MCC950 were obtained from Sigma-Aldrich (St Louis, MO, USA). Fetal bovine serum (FBS) and RPMI-1640 medium were obtained from Gibco (Rockville, MD, USA). oxLDL was purchased from Xiesheng Biotech (Beijing, China). The Cell Counting Kit-8 reagent (CCK-8), Lactate dehydrogenase (LDH) and TUNEL (Fluorescein/TMR red-labeled In-Situ Cell Death) detection kits were obtained from Beyotime (Shanghai, China), Solarbio (Beijing, China) and Roche (Mannheim, Germany), respectively. Anti-NLRP3 (#ab263899) and CD31 (#ab7388) antibodies were obtained from Abcam (Cambridge, MA, USA). Anti-ASC (#67824, #13833), Gasdermin D-N (GSDMD-N) (#36425, #10137), pro-IL-1β (#83186), IL-1β (#12703, #31202), Nrf2 (#12721), HO-1 (#43966), NQO1 (#62262) and Histone H3 (#4499) antibodies were obtained from Cell Signaling Technology (Danvers, MA, USA). Anti-β actin (#A2228), pro-Caspase-1 and anti-Caspase-1(P20) (#AB1871) antibodies were obtained from Sigma-Aldrich (St Louis, MO, USA). Alexa Fluor 488/594-conjugated secondary antibodies were purchased from Invitrogen Life Technologies (Eugene, OR, USA). ELISA Detection Kits for IL-18 and IL-1β were purchased from Enzyme-linked Biotechnology (Shanghai China). 2’,7’-dichlorofluorescein diacetate (DCHF-DA) were purchased from Boshide (Wuhan, China). Malondialdehyde (MDA) and superoxide dismutase (SOD) were obtained from Jiancheng Biotech (Nanjing, China). Finally, the substrates of enhanced chemiluminescence (ECL) were purchased from Thermo Scientific Pierce (Rock-ford, IL, USA).

### Animal experiments

The study was approved by the Laboratory Animals Ethics Committee of Shandong First Medical University. All animal care and experimental procedures complied with the guidelines approved by the Laboratory Animals Ethics Committee of Shandong First Medical University. The experiments were conducted in accordance with the ARRIVE guidelines. Apo e^−/−^ mice (male, six-week-old) on a C57BL/6J background were purchased from Huafukang Bio-Technology Company (Beijing, China). Twenty four Apo e^−/−^ mice were received a high fat diet (15.8% fat and 1.25% cholesterol) for 8 weeks. Thereafter, the mice were randomly allocated to two groups (n = 12 in each group) and administered by gavage for an additional 14 weeks: Pin group (20 mg/kg/day, Pin was dissolved in honey) and model group (the same dosage of honey). Twelve male C57BL/6 mice (6-week-old) were maintained on normal chow diet as a background control group. At the end of the experiment, the mice were sacrificed and the blood samples were collected from their inner canthus and then the hearts, including the aortic roots, were removed transversely. Subsequently, the hearts were embedded in optimum cutting temperature (OCT) compound and then were serially sectioned at 8 μm for the morphological analysis. Additionally, the aortic arches were collected and soaked in liquid nitrogen before being transferred into a −80 °C refrigerator for WB analysis.

### Morphological staining of animal

To assess atherosclerotic lesions, hematoxylin and eosin (H&E) staining was performed according to the manufacturer's protocols and the lesions were captured as digital images using a microscope (Olympus, Tokyo, Japan). The mean lesion area for each mouse was quantified from eight sections using Image-Pro Plus software (version 6.0; Media Cybernetics, MD, USA).

To evaluate the lipid and collagen content in atherosclerotic plaques, continuous frozen sections of aortic roots were stained with oil red O and bright green staining as well as Masson staining, respectively. Morphology staining was photographed using a microscope (Olympus, Tokyo, Japan), and average lipid and collagen content were calculated using Image-Pro Plus software (version 6.0; Media Cybernetics, MD, USA) quantitative analysis.

### Cell culture

HUVECs were obtained from the Type Culture Collection of the Chinese Academy of Sciences (Shanghai, China). Cells were cultured in RPMI-1640 medium supplemented with fetal bovine serum (FBS, 10%) and pen/strep (1%) in a 5% CO_2_ incubator at 37 °C. The medium was replaced with 0.5% FBS medium to starve the cells before experiments.

### CCK-8 and LDH assays

HUVECs were seeded into 96-well plates. After different treatments, cells were incubated with 10% (V/V) CCK-8 reaction solution for 2 h. Optical densities (ODs) were analyzed with a microplate reader (Spectramax i3x, USA) at 450 nm. The viability was described with a percentage of the control condition.

The LDH released into the culture medium was assayed to evaluate HUVECs injury through a specific LDH detection kit that was performed according to instructions from the manufacturer.

### Quantitative real-time PCR

2 μg total RNA from the treated cells was extracted using Trizol reagent (Invitrogen) and was reversely tanscripted using QuantScript 1st strand cDNA synthesis kit (Tiangen Biological Chemistry, Beijing, China). Real-time PCR was carried out on a Rotor-Gene Q real-time PCR cycler (Qiagen, Shanghai, China) with SYBR-green PCR master mix kits. The data were analyzed using the Rotor-Gene Q software (version 1.7, Qiagen), and then relative mRNA levels were calculated using the formula 2^–△△Ct^ method and normalized against GAPDH. The primers used in this study were synthesized by Sangon Biotech (Shanghai, China) and the sequences were as follows: 5′-CAGTCAGCGACGGAAAGAGTA-3′ (forward primer) and 5′-TGTGGGCAACCTGGGAGTAG-3′ (reverse primer) for Nrf2, 5′-GGCAGAGGGTGATAGAAGAGG-3′ (forward primer) and 5′-AGCTCCTGCAACTCCTCAAA-3′ (reverse primer) for HO-1, 5′-CAGCTCACCGAGAGCCTAGT-3′ (forward primer) and 5′-GAGTGAGCCAGTACGATCAGTG-3′ (reverse primer) for NQO1, 5′-GCACCGTCAAGGCTGAGAAC-3′ (forward primer) and 5′-GGATGCAGGGATGATGTTCT-3′ (reverse primer) for GAPDH.

### SiRNA transfection

After transfection with 80 nM specific siRNA directed against Nrf2 and control siRNAs by Lipofectamine 2000 transfection reagent (Invitrogen, Carlsbad, CA, USA), HUVECs were cultured for 48 h. Subsequently, cells were administered with Pin (20 μM, 2 h), followed by the exposure to oxLDL (100 mg/L, 24 h). Western blotting (WB) was carried out to observe the efficiency of Nrf2-siRNA protein knockdown.

### Reactive oxygen species (ROS) detection

DCHF-DA is a reliable fluorescent marker of ROS that becomes oxidized by intracellular ROS to 2', 7'-dichlorofluorescein (DCF) and is used for the detection of intracellular ROS levels. HUVECs with different treatments in 6-well plates were incubated with DCHF-DA (Sigma, 10 μM, 30 min, 37 °C) after being washed in PBS. Green fluorescence signal was detected with a fluorescence microscope (Nikon, Ti-s, Japan) at the excitation of 488 nm and the emission of 525 nm. The mean fluorescence intensity was obtained using the FAC Scan flow cytometer (Becton–Dickinson, San Jose, CA, USA).

### Lipid peroxidation levels and detection of antioxidant enzyme activity

Total protein from treated HUVECs was obtained using the protein extraction kits, and then the protein levels were determined. MDA levels and SOD activity were detected by the corresponding commercial kits as per manufacturer instructions.

### Enzyme-linked immunosorbent assay (ELISA) assay

Fasting blood samples from Apo e^−/−^ mice were obtained. IL-18 and IL-1β levels in serum and medium were tested by ELISA Detection Kits according to the instructions as provided by the manufacturer.

### DNA damage assay

Aortic roots were first incubated with the antibody against CD31 (Abcam, 1:50) for 4 h followed by incubation with donkey anti-rabbit IgG labeled with Alexa Fluor 594 (Invitrogen, 1:1000) for 30 min at room temperature under dark conditions. Treated HUVECs on glass coverslips placed in six-well plates were fixed with paraformaldehyde (4%, 20 min). After permeabilization with Triton X-100 (0.1%, 2 min), the aortic roots and cells were incubated with the TUNEL reaction mixture (Fluorescein for tissues and TMR red for cells) in the dark (1 h) and then stained with DAPI. The images of the samples were captured by an Olympus BX53 fluorescence microscope (Tokyo, Japan) and the percentage of TUNEL or CD31-TUNEL positive cells was measured using the Image-Pro Plus software (version 6.0, Media Cybernetics, LP, USA). The endothelial cells with DNA damage were calculated as the percentage of the number of TUNEL-positive cells to the total HUVECs or CD31-TUNEL positive cells to the plaque areas in the aortic roots.

### WB assay

Protein extraction kits were used to extract nuclear and total proteins from the treated HUVECs and aortic arches as per manufacturer instructions. Protein (40 μg) were separated by sodium dodecyl sulfate polyacrylamide gel electrophoresis (SDS- PAGE) and transferred onto polyvinylidene difluoride (PVDF) membranes. After incubation with rabbit primary antibodies against NLRP3 (1:500), ASC (1:200), GSDMD-N (1:200), pro-Caspase-1 (1:500), Caspase-1(P20, 1:200), pro-IL-1β (1:200), IL-1β (1:200), Nrf2 (1:500), HO-1 (1:200), NQO1 (1:200), Histone 3 (1:500) and β-actin (1:1000) overnight (4 °C), the blots were further incubated with goat anti-rabbit IgG-HRP-labeled secondary antibody for 2 h (room temperature). Bands were visualized by an ECL reaction. The Image-Pro Plus software (ver. 6.0, Media Cybernetics, LP, USA) was used to quantify band intensities, which were normalized to β-actin or Histone 3 levels.

### Immunofluorescence staining

After being washed in PBS, fixated with paraformaldehyde (4%) and permeabilization with Triton-X-100 (0.1%), HUVECs grown on glass coverslips were blocked with normal donkey serum and incubated overnight with anti-Nrf2 (1:50) or anti-Caspase-1 (P20, 1:100) antibodies at 4 °C. Cells were washed and incubated with an Alexa Fluor 488 donkey secondary antibody for 30 min, and were counterstained with DAPI. Nrf2 nuclear translocation and Caspase-1 accumulation in the cytoplasm were observed and captured using an Olympus BX53 fluorescence microscope (Tokyo, Japan).

Serial aortic root cryosections were first blocked with normal donkey serum and then incubated with primary antibodies against CD31 (1:200), NLRP3 (1:200), and Caspase-1 (p20, 1:100) overnight at 4 °C. Then, the frozen sections were treated with Alexa Fluor 594-conjugated donkey anti-rat secondary antibody (1:500) or Alexa Fluor 488-conjugated donkey anti-rabbit secondary antibody (1:500) for 30 min and then stained with DAPI at room temperature. Subsequently, images were captured and digitally analyzed using an Olympus BX53 fluorescence microscope (Tokyo, Japan). Finally, the average fluorescence intensity of the atherosclerotic plaques was determined using Image-Pro Plus software (version 6.0; Media Cybernetics, MD, USA).

### Statistical analysis

Data were described with mean ± standard deviation (SD) for three different experiments at least. Statistical analysis was carried out by one-way analysis of variance (ANOVA) with a Student–Newmann–Keuls test for multiple comparisons and Student’s t test for comparison between two groups in the SPSS19.0 software for Windows. *p* < 0.05 was used to establish statistical significance (Supplementary information).

## Supplementary Information

Supplementary Figures.

## Data Availability

The data presented in the current study are available from the corresponding author on reasonable request.
