# Supplementary material for: Pinocembrin suppresses oxidized low-density lipoprotein-triggered NLRP3 inflammasome/GSDMD-mediated endothelial cell pyroptosis through an Nrf2-dependent signaling pathway
Source: Sci Rep. 2022 Aug 16;12:13885. doi: 10.1038/s41598-022-18297-3 (PMC9381505; doi:10.1038/s41598-022-18297-3)

Pinocembrin suppresses oxidized low-density lipoprotein-triggered NLRP3 inflammasome/GSDMD-mediated endothelial cell pyroptosis through an Nrf2-dependent signaling pathway

Tong Wang[1§], Hua Tian[2,3§✉], Tianqi Pan[4], Shutong Yao[4], Huayun Yu[2], Yumei Wu[5] & Shijun Wang[2✉]

[1] College of nursing, Shandong University of Traditional Chinese Medicine, Jinan 250355, Shandong, China.

[2] College of Traditional Chinese Medicine, Shandong University of Traditional Chinese Medicine, Jinan 250355, Shandong, China.

[3] Key Laboratory of Atherosclerosis in Universities of Shandong and Institute of Atherosclerosis, Shandong First Medical University & Shandong Academy of Medical Sciences, Taian 271000, Shandong, China.

[4] College of Basic Medical Sciences, Shandong First Medical University & Shandong Academy of Medical Sciences, Taian 271000, Shandong, China.

[5] Pharmacy Department, Dongying district hospital of Traditional Chinese Medicine, Dongying 257000, Shandong, China.

[§] Equal contribution to this study

[✉] To whom correspondence should be addressed:

[1] Shijun Wang, Email: wsj@sdutcm.edu.cn, [2] Hua Tian, Email:liutianfangyu @163.com, College of Traditional Chinese Medicine, Shandong University of Traditional Chinese Medicine, No.4655 Daxue Road, Changqing District, Jinan 250355, Shandong, China. Tel.: +86 0531 89628750; Fax: +86 0531 89628077.

Figure 1c

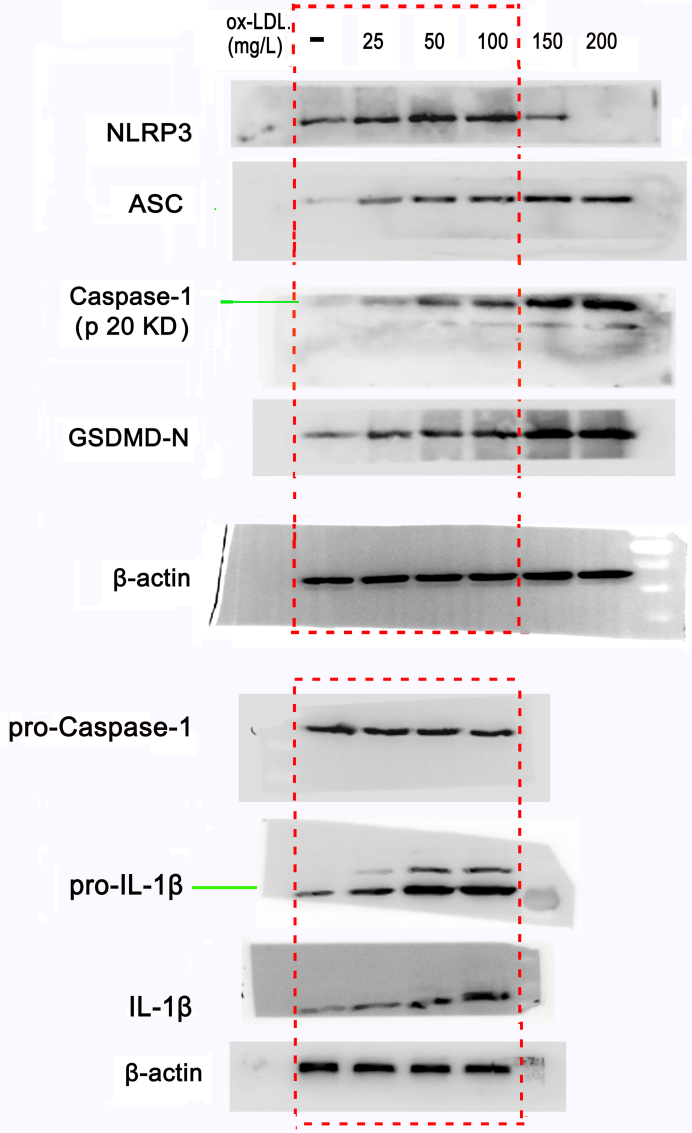

Supplementary data

Supplementary data

Supplementary data

Supplementary data

Figure 3 a

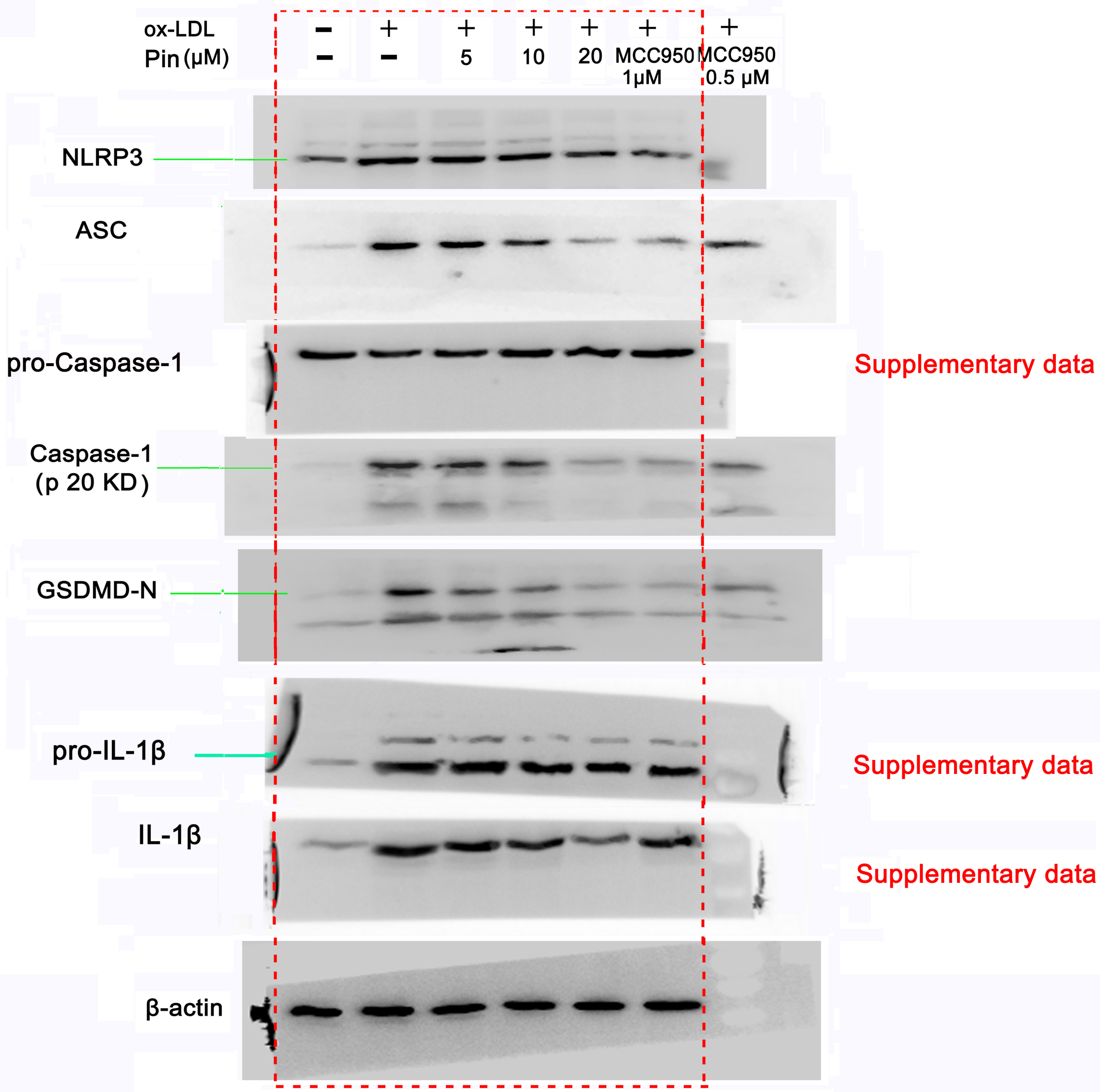

ox-LDL
Pin (μM)
− − + − + 5 + 10 + 20 + MCC950 1μM + MCC950 0.5 μM
NLRP3
ASC
pro-Caspase-1
Caspase-1 (p 20 KD)
GSDMD-N
pro-IL-1β
IL-1β
β-actin
Supplementary data
Supplementary data
Supplementary data

Figure 4 a

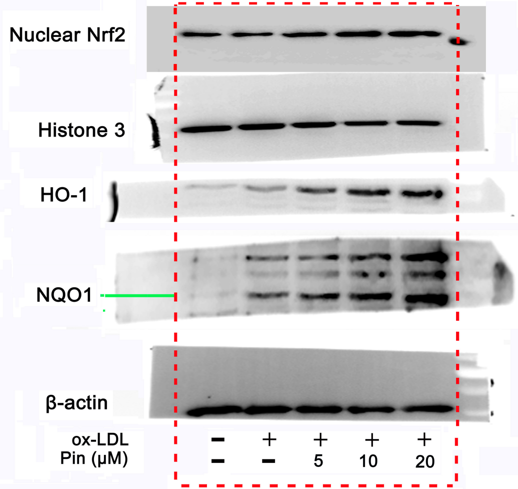

Figure 4 c

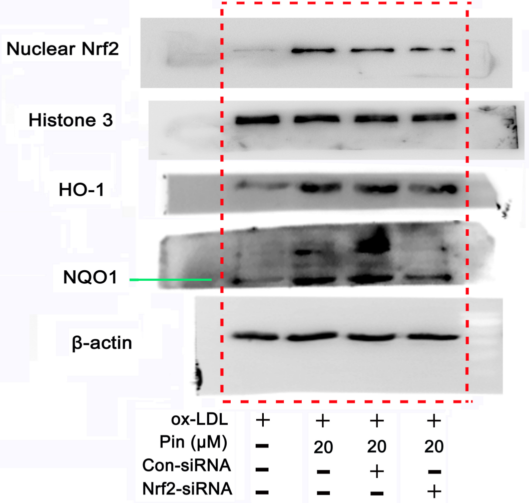

Figure 6 b

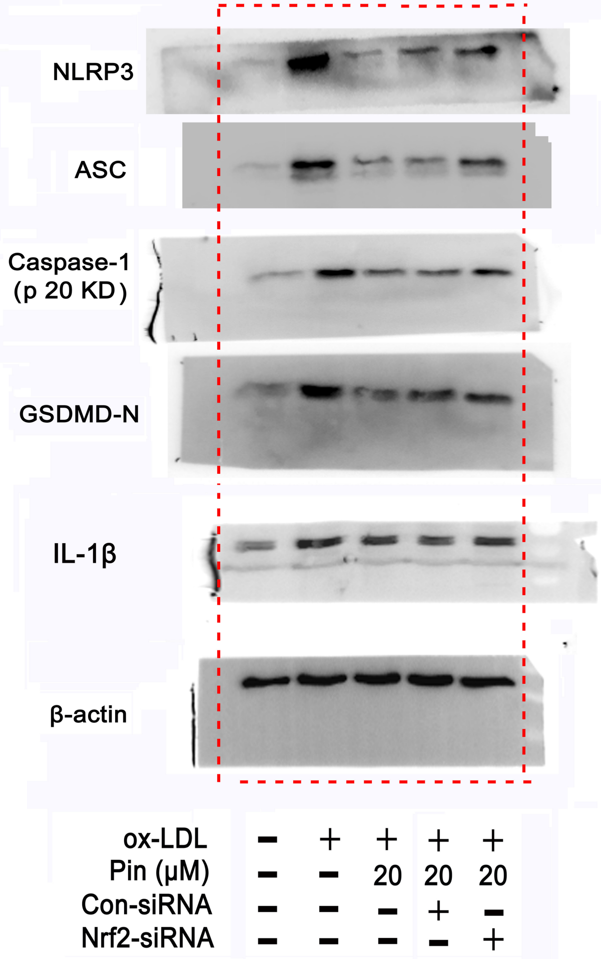

Supplementary data

Figure 8 d

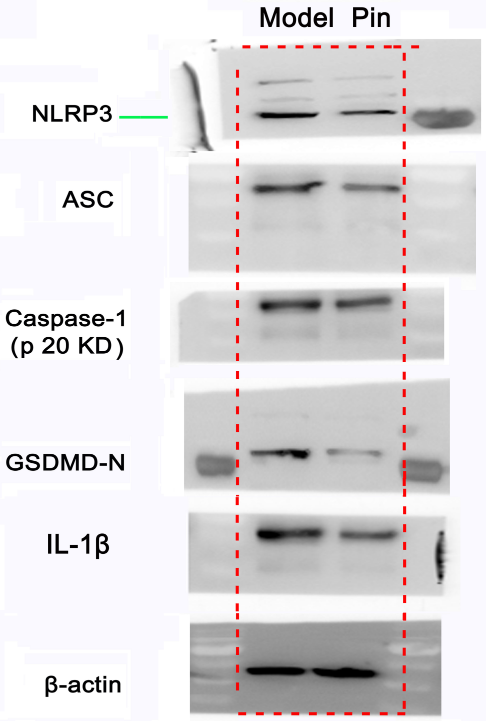

Figure 8 e

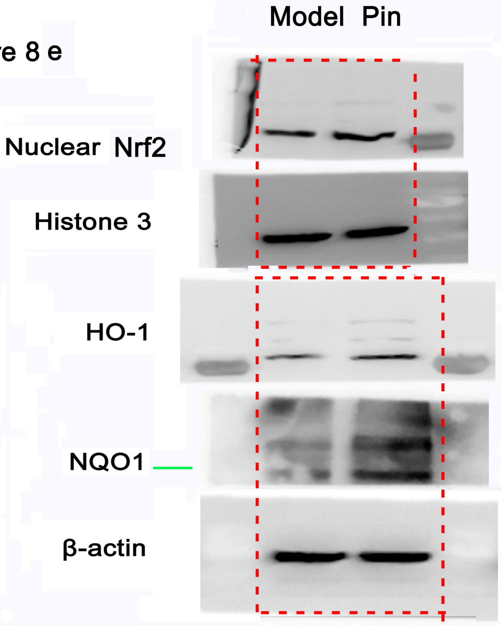

Supplement: Supplementary file 1 — Supplementary Figures. [file 41598_2022_18297_MOESM1_ESM.pdf]
